# Calibration of an orientation sensor for freehand 3D ultrasound and its use in a hybrid acquisition system

**DOI:** 10.1186/1475-925X-7-5

**Published:** 2008-01-24

**Authors:** Richard James Housden, Graham M Treece, Andrew H Gee, Richard W Prager

**Affiliations:** 1 Department of Engineering, University of Cambridge, Trumpington Street, Cambridge CB2 1PZ, UK

## Abstract

**Background:**

Freehand 3D ultrasound is a powerful imaging modality with many potential applications. However, its reliance on add-on position sensors, which can be expensive, obtrusive and difficult to calibrate, is a major drawback. Alternatively, freehand 3D ultrasound can be acquired without a position sensor using image-based techniques. Sensorless reconstructions exhibit good fine scale detail but are prone to tracking drift, resulting in large scale geometrical distortions.

**Method:**

We investigate an alternative position sensor, the Xsens MT9-B, which is relatively unobtrusive but measures orientation only. We describe a straightforward approach to calibrating the sensor, and we measure the calibration precision (by repeated calibrations) and the orientation accuracy (using independent orientation measurements). We introduce algorithms that allow the MT9-B potentially to correct both linear and angular drift in sensorless reconstructions.

**Results:**

The MT9-B can be calibrated to a precision of around 1°. Reconstruction accuracy is also around 1°. The MT9-B was able to eliminate angular drift in sensorless reconstructions, though it had little impact on linear drift. In comparison, six degree-of-freedom drift correction was shown to produce excellent reconstructions.

**Conclusion:**

Gold standard freehand 3D ultrasound acquisition requires the synthesis of image-based techniques, for good fine scale detail, and position sensors, for good large scale geometrical accuracy. A hybrid system incorporating the MT9-B offers an attractive compromise between quality and ease of use. The position sensor is unobtrusive and the system is capable of faithful acquisition, with the one exception of linear drift in the elevational direction.

## Background

3D ultrasound [1,2] is an emerging medical imaging modality with a wide range of potential applications [3]. The data can be acquired using dedicated 3D probes incorporating either a 2D array or an oscillating head which sweeps the B-scan plane over a fixed volume. The alternative, freehand approach involves the clinician manually sweeping a conventional probe over the target: by attaching a position sensor to the probe, each B-scan can be labelled with its position and orientation. The B-scans thus form a 3D data set which can be visualised and processed in a number of ways to extract clinically useful information. The freehand approach offers the advantages of arbitrary acquisition volumes, with translation as well as rotation of the scan head, low cost, and compatibility with the full palette of existing 2D probes. There are also disadvantages, including slow acquisition: freehand acquisition is not suitable for 4D scanning.

The 2D array and oscillating head approaches are the current focus of commercial activity. Perhaps the greatest barrier to more widespread uptake of freehand scanning is the add-on position sensor. Most position sensors for freehand 3D ultrasound fall into two categories: optical sensors, which employ two or more cameras to track targets attached to the probe, and magnetic sensors, which use a small receiver mounted on the probe to measure a spatially varying magnetic field generated by a fixed base station. Both types of sensor require careful calibration [4] and impose constraints on the scanning protocol. For optical position sensors, the user must maintain a clear line of sight between the probe and the cameras, and must be careful not to stray outside the cameras' field of view. Magnetic sensors also suffer from a limited operating region: furthermore, the immediate vicinity must be kept clear of metallic objects and stray magnetic fields. 3D reconstructions based on position sensor readings also suffer from fine scale jitter artefacts. The jitter arises through a combination of noisy sensor readings and misregistration caused by small probe pressure distortions of the anatomy. As such, it is not possible to completely remove the jitter simply by processing the sensor readings.

It is against this background that we evaluate an unconventional sensor that has received no attention from the 3D ultrasound community. The Xsens MT9-B [5] uses MEMS magnetometers, accelerometers and rate gyros to determine its orientation. MEMS devices are extremely compact and could easily be built into a probe assembly without inconveniencing the user. There is no line of sight requirement, no restricted operating region, just a moderate sensitivity to proximate ferromagnetic materials, although this need not be an issue in typical clinical practice with appropriate consideration of the scanning environment. The obvious limitation is that the MT9-B is a three degree-of-freedom device, measuring orientation but not position. How such a device might be useful for freehand 3D ultrasound acquisition will be explained shortly.

**Figure 1:**
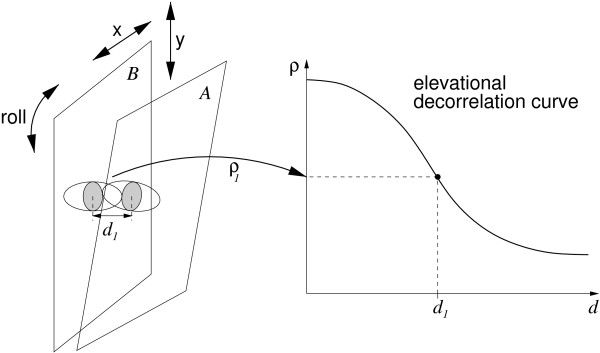
**Principle of elevational speckle decorrelation**. The in-plane motion between scans *A *and *B *(translation in the *x *and *y *directions, roll around the plane normal) is readily determined using conventional 2D image registration techniques. This leaves three degrees of freedom: translation in the elevational direction, tilt (rotation about *x*) and yaw (rotation about *y*). Consider corresponding patches in scans *A *and *B *(the shaded ellipses). Because of the imperfect elevational focusing, the contents of the patches depend on scatterers within overlapping resolution cells (the hollow ellipsoids) and are therefore correlated. The correlation coefficient depends on the degree of overlap and hence the elevational separation. It follows that, given a suitable decorrelation curve, a measured correlation *ρ*_1 _can be used to look up the corresponding separation *d*_1_. Repeating this process for three (or more) non-collinear patches determines the out-of-plane separation, tilt and yaw of *A *relative to *B*.

Freehand 3D ultrasound can also be acquired, without a position sensor, by deducing the probe's motion from the B-scan images themselves. Consider two neighbouring B-scans *A *and *B *in a freehand sequence. Any in-plane motion between *A *and *B *(translation in the axial and lateral directions, roll around the elevational axis) is readily determined using standard 2D image registration techniques [6,7]. Perhaps surprisingly, the out-of-plane motion components can also be estimated from the images [8-10]. This is because the focusing of the ultrasound beam is far from perfect. Consequently, the backscattered signal at any point in a B-scan is a function of the scatterers in a certain *resolution cell *around that point. The resolution cells are particularly elongated in the elevational direction and there is considerable spatial overlap between cells on neighbouring B-scans – see Figure 1. The echo signals in corresponding patches on *A *and *B *are therefore correlated, with the degree of correlation depending on the patches' elevational separation. The correlation between three (non-collinear) pairs of patches can therefore be used to infer the three patch separations and hence the out-of-plane separation, tilt and yaw of *A *relative to *B*.

In our own recent work on sensorless freehand 3D ultrasound, we have extended its capabilities to encompass arbitrary tissue types [11] and arbitrary probe motion [12,13]. Compared with position sensor-based alternatives, sensorless reconstructions exhibit superior fine scale detail [13]. However, it is difficult to eliminate all sources of bias from the elevational offset estimates. Consequently, there is a cumulative drift error as the inter-frame displacements are concatenated to build up the overall 3D reconstruction. It is at this point that we return to our earlier discussion of the MT9-B orientation sensor. Clearly, this device could be used to correct any angular drift in sensorless reconstructions. Less obviously, it has the potential to ameliorate linear drift too, by filtering out incorrect elevational separation estimates that are inconsistent with the measured orientation.

In the following sections, we investigate the MT9-B with this drift-correction application in mind. We describe its calibration and measure the calibration precision and the reconstruction accuracy. Finally, we describe how orientation measurements can be used to reduce drift in sensorless reconstructions.

## Methods

### Calibrating the MT9-B position sensor

The MT9-B was mounted on a 5–10 MHz linear array probe connected to a Dynamic Imaging Diasus [14] ultrasound machine. The depth setting was 4 cm with a single focus at 2 cm. Analogue RF ultrasound signals were digitised after receive focusing and time-gain compensation, but before log-compression and envelope detection, using a Gage Compuscope CS14200 14-bit digitiser [15]. Sampling was at 66.67 MHz, synchronous with the ultrasound machine's internal clock: this synchronisation minimises phase jitter between vectors. The acquired vectors were filtered with a 5–10 MHz filter, then envelope-detected using the Hilbert transform. Each B-scan comprises 127 vectors, with 3818 samples per vector. The resolution is approximately 0.01 mm per sample in the axial direction and 0.3 mm per vector in the lateral direction.

Knowing the MT9-B's orientation is quite different to knowing the B-scan's orientation. To relate the two, we need to determine the three degree-of-freedom rotation matrix between the B-scan and MT9-B coordinate systems. This is a restricted version of the well-known spatial calibration problem for six degree-of-freedom position sensors. While there are many techniques for solving the six degree-of-freedom problem [16], most are not suitable for our restricted, orientation-only problem, since the rotation parameters are not adequately decoupled from the translation parameters.

One technique which does lend itself to the restricted problem is the plane-based calibration technique of [17]. This involves scanning a flat plane at the bottom of a water bath, producing a straight-line echo in the B-scan. For the full, six degree-of-freedom calibration problem, there are eleven unknowns: six for the desired rigid body transformation between the sensor and B-scan coordinate systems, three for the position and orientation of the scanned plane, and two image scale parameters. The scale parameters are determined separately using the ultrasound scanner's length measurement facility [18]. For the remaining nine unknowns, each image of the plane, coupled with the corresponding reading from the position sensor, provides two constraints. Provided the probe is moved in such a way as to exercise all degrees of freedom [19], a set of images and sensor readings provides sufficient constraints to solve for these position and orientation unknowns. [20] describes a three-stage nonlinear optimisation process for determining the solution. Significantly, the first stage solves for the rotation parameters using only the orientation readings from the position sensor. This is precisely what is required to calibrate the MT9-B sensor.

**Figure 2:**
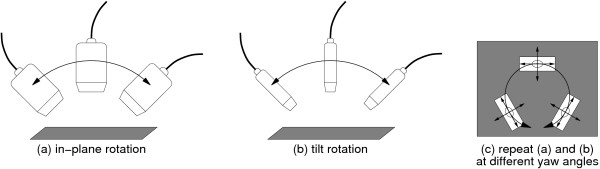
**Plane-based calibration protocol for the MT9-B**. Compared with six degree-of-freedom sensors [19], a simpler set of probe motions suffices to solve for the calibration parameters.

Since there are only five degrees of freedom at this stage (three for the orientation of the MT9-B with respect to the B-scan, and two for the orientation of the plane), a simpler set of probe motions provides the necessary constraints, as illustrated in Figure 2. With this one exception, we adopt the same calibration protocol as in [19], including the use of a "Cambridge phantom" to produce echoes from a flat plane without sensitivity to beam-width effects.

Given only orientation data, there is one ambiguity in the calibration solution. It is possible to rotate the B-scan 180° in-plane, this "mirror" solution remaining consistent with the MT9-B readings and the orientations of the echo lines in the B-scans. We overcome this problem by making use of the known geometry of the experimental setup. The coordinate system in which the MT9-B provides its readings is not arbitrary. The *x *direction is aligned with magnetic north and the *z *direction is upwards, defined by gravity. In addition, the calibration plane is roughly horizontal and viewed from above. The average B-scan axial direction should therefore have a negative *z *component in MT9-B coordinates. By checking whether this is the case, we can resolve the ambiguity and correct the calibration if necessary.

### Assessing calibration precision and reconstruction accuracy

The calibration precision was assessed by repeating the calibration process 25 times and calculating the standard deviations of the three significant components of the solution (the orientation of the B-scan with respect to the MT9-B in terms of roll, yaw and tilt). At each repetition, the probe was removed and remounted in the Cambridge phantom.

Reconstruction accuracy was assessed by mounting the probe and sensor assembly on a mechanism that could be set accurately to known positions and orientations [21]. The mechanism allows rotation about two axes, and was set to eleven tilt values in the range -16.4° to +2.2° from the vertical, and seven yaw values in the range -5.5° to +5.5°, giving 77 orientations in total (the angular ranges were limited by the design of the mechanism). Since the probe was attached to the mechanism in such a manner as to align the B-scan approximately with two of the mechanism's principal axes, the 77 measured orientations were readily converted to B-scan orientations.

These B-scan orientations were also obtained from the calibrated MT9-B readings. The rotation between every pair of frames from the 77 was calculated, using each of the 25 MT9-B calibrations, giving a total of 73,150 inter-frame orientations. Each orientation was expressed in terms of rotations about the frame pair's average *x*, *y *and *z *axes, which we refer to as tilt, yaw and roll respectively. The MT9-B's accuracy was assessed in terms of the mean and standard deviation of the errors in these angles, using the corresponding mechanism readings as ground truth.

This experiment was performed with the probe mounted in two orientations on the mechanism. For the first experiment, the probe was aligned so that tilt and yaw of the mechanism was approximately equivalent to tilt and yaw of the B-scan. The probe was then remounted at approximately 90° to its original orientation, so that tilting the mechanism produced roll in the B-scan. This second mounting also brought the sensor closer to a ferromagnetic component of the mechanism, allowing some assessment of the MT9-B's magnetic sensitivity.

### Sensorless drift correction using orientation measurements

**Figure 3:**
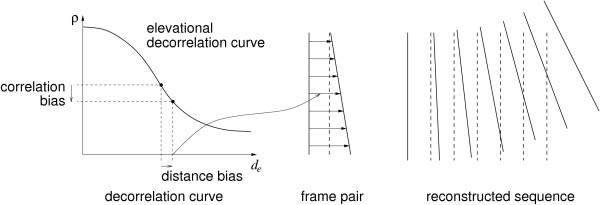
**Reconstruction drift**. The sensorless reconstruction paradigm assumes that the correlation between two patches of data depends only on the separation between them. However, various other effects can bias the correlation value. This results in biased inter-frame offsets and a consequential drift error in the reconstructed sequence. Here, the dashed lines show the correct frame positions and the solid lines show the drifted reconstruction.

While the MT9-B is unobtrusive, it measures only orientation and is therefore not suitable for use in a position sensor-based freehand 3D ultrasound system. However, it could be used to reduce drift errors in sensorless acquisition systems. Figure 3 illustrates the two predominant drift errors, namely stretch and tilt [13]. Speckle decorrelation occurs for reasons other than elevational separation, including probe rotation, physiological motion, probe pressure-induced deformation and electrical noise. The reduced correlation between B-scan patches is misinterpreted as increased elevational separation, leading to an erroneously stretched reconstruction in the elevational direction. Furthermore, there is a depth-dependent component of the bias, resulting in angular drift in the tilt direction, as illustrated in Figure 3.

In describing how the MT9-B might be used to ameliorate these drift errors, and assessing its efficacy in this role, it is informative to begin with six degree-of-freedom drift correction using a six degree-of-freedom position sensor. In the sensorless reconstruction, the position of each frame *f *is expressed as a homogeneous transformation matrix **S**_*f*_. We correct these positions by pre-multiplying **S**_*f *_by **T**_*f*_, where

$$T_{f}=\{\begin{array}{lll} T_{f-1}S_{f}C_{f}S_{f}^{-1} & \text{for} & f\geq 1\\ S_{f}C_{0}S_{f}^{-1} & \text{for} & f=0 \end{array}$$

The correction **C**_*f*_, relative to the previous frame in the sequence, is most naturally expressed in B-scan coordinates (since the various sources of bias depend on B-scan depth). Multiplying by **T**_*f*-1 _takes account of all the adjustments made to earlier frames in the sequence.

**C**_*f *_is defined as a function of the six parameters (three translations and three rotations) specifying the six degree of freedom adjustment as follows:

$$C_{f}(x,y,z,\theta ,\phi ,\psi )=\left(\begin{array}{cccc} \cos\phi \cos\psi & \sin\theta \sin\phi \cos\psi -\cos\theta \sin\psi & \cos\theta \sin\phi \cos\psi +\sin\theta \sin\psi & x\\ \cos\phi \sin\psi & \sin\theta \sin\phi \sin\psi +\cos\theta \cos\psi & \cos\theta \sin\phi \sin\psi -\sin\theta \cos\psi & y\\ -\sin\phi & \sin\theta \cos\phi & \cos\theta \cos\phi & z\\ 0 & 0 & 0 & 1 \end{array}\right)$$

where *x*, *y *and *z *define the translation, and *θ*, *φ *and *ψ *are the rotations about the *x*, *y *and *z *axes respectively. The six parameter values are determined using a set of six cubic curves (two are shown here):

$$\begin{array}{c} x=\alpha_{0}+\alpha_{1}t+\alpha_{2}t^{2}+\alpha_{3}t^{3}\\ y=\beta_{0}+\beta_{1}t+\beta_{2}t^{2}+\beta_{3}t^{3}\\ \vdots \end{array}$$

where *t *is a value between 0 and 1, defining how far through the sequence the frame is. The cubic formulation ensures that the drift correction is smoothly varying along the sequence, and cannot over-fit to noise in the position sensor readings. We also allow an initial offset at the first frame, defined by an additional six parameters, independent of the drift correction in (3). The overall effect can be thought of as a rigid registration of the sensorless sequence to the position sensor readings (the additional six parameters), combined with the non-rigid deformation in (3).

The coefficients of Equation (3) and the six initial offset parameters are determined by nonlinear optimisation (Levenberg-Marquardt [22]) so that the adjusted sensorless reconstruction is as close as possible to the position sensor measurements, subject to the smoothness constraint implicit in (3). The optimisation error function is

$$\sum_{f}\sum_{c}\left|T_{f}S_{f}w_{c}-P_{f}w_{c}\right|$$

where **P**_*f *_is the position of frame *f *measured by the position sensor and **w**_*c *_defines the location of frame corner *c *in B-scan coordinates. The total error is therefore the sum of absolute distances between the drift-corrected sensorless positions (**T**_*f *_**S**_*f*_) and the measured positions (**P**_*f*_), at each of the four corners of the B-scan. This ensures that errors in the B-scans' orientations, as well as their positions, are detected and minimised.

For the MT9-B, a restricted, three degree-of-freedom correction is feasible. Instead of the six correction curves in (3) and the six initial offset parameters, we have just three of each. The error function for the nonlinear optimisation is then

$$\sum_{f}\left(\text{roll}{\left(T_{f}S_{f},P_{f}\right)}^{2}+\text{yaw}{\left(T_{f}S_{f},P_{f}\right)}^{2}+\text{tilt}{\left(T_{f}S_{f},P_{f}\right)}^{2}\right)$$

This is the sum of the squared roll, yaw and tilt of the drift corrected sensorless frame positions relative to the measured frame positions. Since we are correcting only orientation, **C**_*f *_is constrained to rotate the B-scan in such a way that its centre does not move relative to the previous B-scan. In the example of Figure 3, this adjustment would decrease the angle between each frame pair, and this alone would move the end frame significantly closer to its correct position.

**Figure 4:**
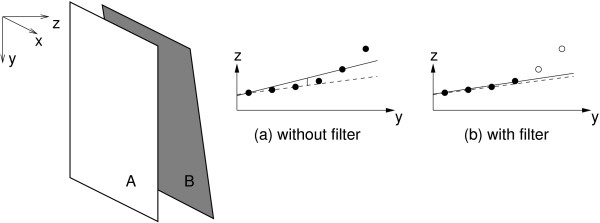
**Filtering incorrect distance estimates**. Biases in the individual distance estimates can lead to biases in the elevational offset and tilt when a plane is fitted to the distances, as shown in (a). The dashed line shows the correct plane and the solid line is the plane suggested by the elevational distance estimates. By removing distance estimates that are not consistent with the measured orientation, the bias can be reduced, as shown in (b).

In addition, there is another way in which orientation measurements might be used to ameliorate linear, stretch drift. The orientation data could support a filter to remove biased elevational distance estimates. Figure 4 shows a typical frame pair and the resulting pattern of distance estimates in the axial direction, as determined by speckle decorrelation. In this example, the bias in the distance estimates is larger towards the bottom of the frame. This could be caused by a lack of signal in this region of the image, producing lower correlations and hence overestimated distance estimates. Fitting a B-scan plane (using least squares) to these distances results in a tilt bias and a small overestimate in the elevational centre offset, as shown in Figure 4(a).

The proposed filter is based on the Random Sample Consensus algorithm [23]. We consider each elevational distance estimate individually and fit a B-scan plane to it using the orientation measured by the MT9-B. We then count how many of the other distance estimates support the hypothesis that this plane is correct. The plane that attracts the largest support provides the set of "correct" distance estimates and any others are then ignored. In the example in Figure 4(b), the distance estimates highlighted would be ignored, resulting in the much better fit shown in the figure. The filter has one tunable parameter: the threshold specifying how close a distance estimate must be to the fitted plane to count as a "supporter". This is set to one standard deviation of the estimated uncertainty in the distances, determined using the method described in [13].

Drift correction was assessed by freehand scanning of a speckle phantom (from the Department of Medical Physics, University of Wisconsin, Madison, Wisconsin, USA) and animal tissue *in vitro*. The sensorless reconstructions were obtained using the technique described in [13] and ground truth was available from a Northern Digital Polaris [24] optical position sensor tracking a Traxtal AdapTrax [25] target attached to the probe. Temporal calibration was performed according to the technique in [19]. For reasons of comparison and algorithm verification, the sensorless reconstructions were first corrected using both position and orientation data from the Polaris, then using just the orientation data, in the manner described above. Note that the MT9-B was not used in these experiments, with good reason: the Polaris provides an essential ground truth against which to assess the orientation-only drift correction. However, we also present a qualitative *in vivo *scan of a human calf muscle corrected using the MT9-B.

## Results and Discussion

### MT9-B calibration precision

Table 1 shows precision statistics for the MT9-B calibration process. The in-plane roll, and the out-of-plane yaw and tilt angles, are expressed in the B-scan reference frame. The results indicate significant dependence on the accuracy of detecting the lines (the echoes of the calibration plane) in the B-scan images. For example, the tilt calibration parameter depends strongly on the measured angles of the echo lines during motion (a) in Figure 2. Unfortunately, when the plane is imaged in this manner it is difficult to obtain a clear echo and measure its orientation reliably. It is therefore not surprising that the worst precision is in the tilt angle. Nevertheless, the calibration precision is comparable to previous results for six degree-of-freedom sensors [19]. Calibration imprecision is one factor affecting the reconstruction accuracy determined in the next section.

**Table 1:** MT9-B calibration precision. The table shows the standard deviation, in degrees, of the in-plane roll, and the out-of-plane yaw and tilt angles, of the 25 calibrations relative to the average calibration.

	**Roll**	**Yaw**	**Tilt**
**Standard deviation (degrees)**	0.08	1.10	1.50

### MT9-B reconstruction accuracy

Table 2 summarises the results of the two accuracy experiments. The tabulated statistics relate to the accuracy of measuring angles between frame pairs and therefore give an indication of the accuracy for drift correction. It is immediately apparent from these results that the yaw accuracy is worse than the roll and tilt accuracy, and is particularly bad in the roll-yaw experiment. This difference is not unexpected, since the roll and tilt measurements depend predominantly on the MT9-B's accelerometer measurements of the gravitational vertical, while the yaw relies more on the magnetometer. In the roll-yaw experiment, the sensor was mounted closer to the field-distorting ferromagnetic material, causing larger errors in the yaw angle.

**Table 2:** MT9-B orientation accuracy. The table shows the orientation reconstruction accuracy in terms of the mean and standard deviation of the error between frame pairs. Values are in degrees.

	**Roll**	**Yaw**	**Tilt**
**Tilt-yaw rotation**	-0.0593 ± 0.3058	-0.4633 ± 0.8887	+0.0641 ± 0.1658
**Roll-yaw rotation**	+0.0371 ± 0.3117	-2.1223 ± 1.8843	-0.2040 ± 0.3092

**Figure 5:**
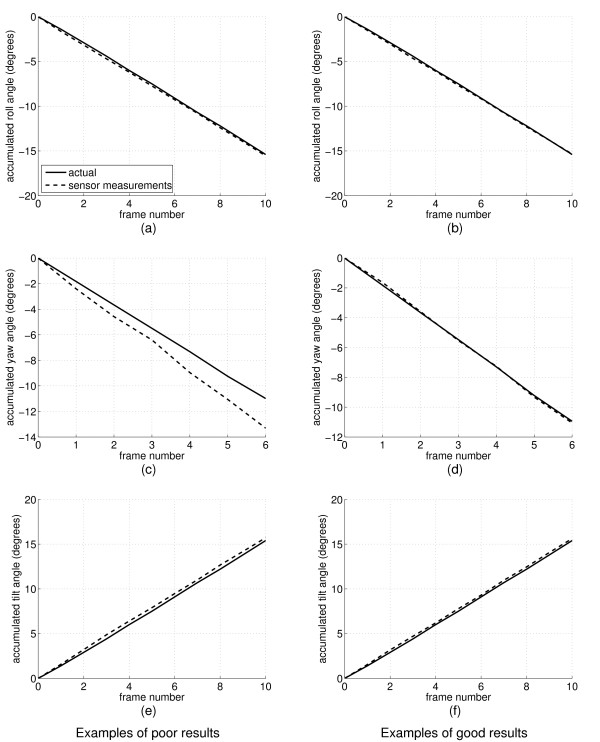
**MT9-B reconstructions**. The graphs show the roll, yaw and tilt measured by the sensor, compared with the same measurements deduced from the controlling mechanism. The left column shows examples of the worst drift in each angle and the right column shows examples of the best. Graphs (a-c) were derived form the roll-yaw experiment, which had some nearby ferromagnetic material. Graphs (d-f) are from the tilt-yaw experiment, where the ferromagnetic material was more distant.

Figure 5 shows some representative examples of the orientations measured using the MT9-B, compared to the actual orientations set by the mechanism. For each of the three rotation axes, the graphs are for a sequence of frames selected from the grid of orientations so that there is no rotation about the other two axes. The most notable feature of these graphs is the yaw drift in the first column of the figure. This is due to the magnetic field distortion caused by the small ferromagnetic parts of the controlling mechanism. The better yaw result in the second column is from the tilt-yaw experiment, where the sensor was mounted further away from the ferromagnetic part. In comparison, the roll and tilt graphs, which do not depend significantly on the MT9-B's magnetometer readings, show no sign of drift.

These results demonstrate that the MT9-B is capable of high accuracy orientation measurement, provided it is kept clear of magnetic field distortions. While this requires careful consideration of the scanning environment, it is not difficult to achieve. For the small amounts of ferromagnetic material present in our case, a few centimetres clearance was sufficient to remove the yaw drift and obtain an accuracy within 1°.

### Sensorless drift correction

**Figure 6:**
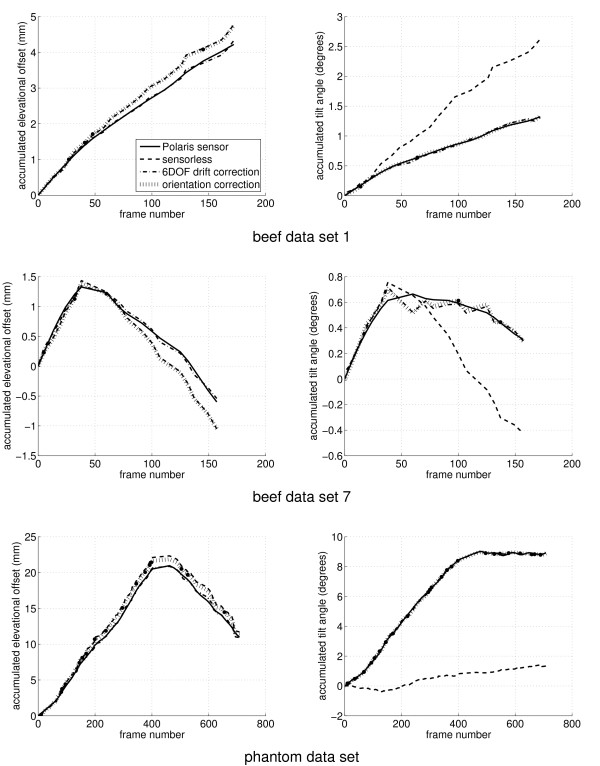
**Drift correction examples**. The graphs show sample reconstructions from the eleven test data sets. The left column shows the elevational offset at the centre of the frame accumulated through the data set. The right column shows accumulated tilt angle.

Figure 6 shows representative results for ten sweeps of *in vitro *animal tissue (a joint of beef) and an additional sweep of a speckle phantom with spherical inclusions. The reconstructions are shown in terms of their accumulated elevational length (left column) and their accumulated tilt (right column). Tables 3 and 4 show numerical results for all eleven data sets. Note the intentional nonmonotonic scanning motion in the second beef graph and the phantom data set. Data sets 1–10 were essentially linear sweeps in the elevational direction, with data sets 6–10 including a deliberate change of direction part way through the sweep. The phantom data set also featured deliberate probe rotation. Each plot in Figure 6 comprises four traces: the Polaris reconstruction, the sensorless reconstruction, and the drift-corrected sensorless reconstructions using six degree-of-freedom and orientation-only correction.

**Table 3:** Drift correction results for length. The table shows values for the error in the elevational length of the reconstructed sequences for the various reconstruction methods. Values are in millimetres, with the values in brackets showing the error as a percentage of the correct overall length (determined from the Polaris sensor). For the nonmonotonic data sets, results are shown separately for the forward and reverse sweeps, as indicated by the *f *and *r *subscripts respectively.

**Data set**	**Image-based**		**Six DOF correction**		**Orientation correction**	
**1**	0.55	(13.1)	0.11	(2.5)	0.53	(12.5)
**2**	0.96	(30.5)	-0.09	(-2.9)	0.99	(31.6)
**3**	0.75	(26.3)	0.00	(0.2)	0.75	(26.5)
**4**	-0.56	(-19.9)	0.05	(1.9)	-0.52	(-18.3)
**5**	-0.16	(-6.8)	0.09	(3.8)	-0.19	(-8.0)
**6**_*f*_	0.28	(14.6)	0.05	(2.9)	0.24	(12.7)
**6**_*r*_	0.21	(15.6)	0.06	(4.61)	0.22	(16.0)
**7**_*f*_	0.11	(8.01)	0.01	(0.6)	0.07	(4.9)
**7**_*r*_	0.56	(29.0)	-0.04	(-2.0)	0.51	(26.7)
**8**_*f*_	0.07	(2.7)	0.05	(2.2)	0.05	(2.0)
**8**_*r*_	-0.18	(-7.6)	-0.02	(-1.0)	-0.16	(-6.5)
**9**_*f*_	0.54	(23.9)	-0.02	(-1.0)	0.58	(25.5)
**9**_*r*_	-0.40	(-21.7)	0.01	(0.7)	-0.43	(-23.4)
**10**_*f*_	0.05	(3.0)	0.02	(1.4)	-0.03	(-1.6)
**10**_*r*_	0.59	(40.9)	-0.00	(-0.1)	0.37	(25.6)
**phantom**_*f*_	1.37	(6.5)	-0.23	(-1.1)	0.89	(4.2)
**phantom**_*r*_	0.61	(6.1)	-0.05	(-0.5)	0.47	(4.7)

**Table 4:** Drift correction results for tilt. The table shows values for the error in the accumulated tilt of the reconstructed sequences for the various reconstruction methods. Values are in degrees. Percentage values are not useful in this case, as the correct tilt angles are very close to zero for the beef data sets. For the nonmonotonic data sets, results are shown separately for the forward and reverse sweeps, as indicated by the *f *and *r *subscripts respectively.

**Data set**	**Image-based**	**Six DOF correction**	**Orientation correction**
**1**	1.30	-0.06	-0.01
**2**	1.50	-0.07	-0.09
**3**	0.46	0.03	0.01
**4**	1.35	0.01	0.00
**5**	1.25	-0.09	-0.03
**6**_*f*_	0.71	-0.03	-0.01
**6**_*r*_	0.61	-0.01	-0.02
**7**_*f*_	0.14	0.05	0.09
**7**_*r*_	0.87	0.11	0.10
**8**_*f*_	0.67	0.01	-0.02
**8**_*r*_	0.53	-0.00	0.01
**9**_*f*_	1.14	0.07	0.05
**9**_*r*_	-0.05	0.07	0.07
**10**_*f*_	1.50	-0.03	-0.02
**10**_*r*_	2.21	0.02	0.03
**phantom**_*f*_	-8.02	0.03	-0.11
**phantom**_*r*_	-0.43	-0.03	0.00

It is evident from the figure and tables that the six degree-of-freedom corrections achieve the goal of producing a geometrically accurate reconstruction. The orientation-only correction achieves the expected elimination of angular drift, but the filtering strategy has had little effect on the linear drift. From this, we can infer that any axial variation in elevational offset bias (this variation being the cause of tilt drift) is evenly distributed about the centre of the B-scan, so correcting the orientation does not affect the centre offset. Consequently, a small bias will remain in any measurements taken from the reconstructed data.

**Figure 7:**
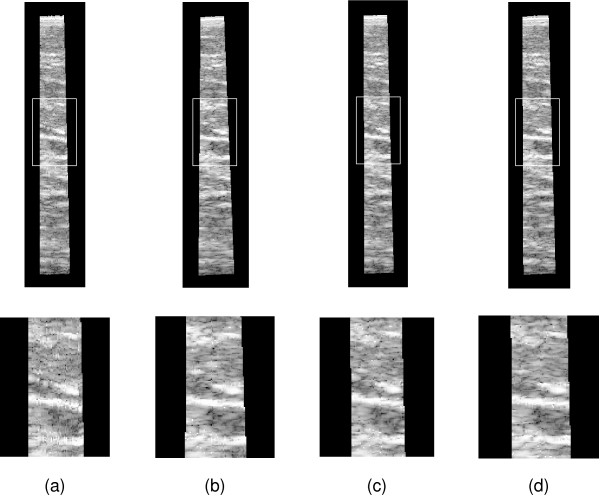
**Reslice images of beef data set 1**. The images show reslices through beef data set 1. The upper row of reslices are along the length of the data, showing the length and tilt. The lower row shows enlarged versions of the outlined region, highlighting the fine scale accuracy. (a) Position sensor only reconstruction. (b) Sensorless only. (c) Six degree-of-freedom correction. (d) Orientation-only correction.

**Figure 8:**
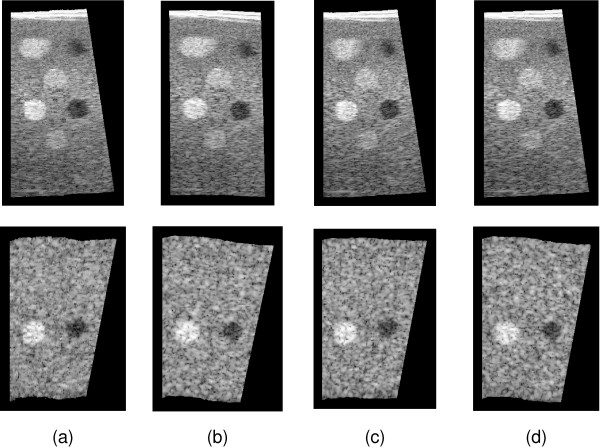
**Reslice images of phantom data**. The images show reslices through the phantom data set. The upper row of reslices are along the length of the data, showing the length and tilt. The lower reslices are parallel to the scanning surface and therefore show length and yaw. All the reslices use frames from the forward sweep only. (a) Position sensor only reconstruction. (b) Sensorless only. (c) Six degree-of-freedom correction. (d) Orientation-only correction.

Figures 7 and 8 show reslices though beef data set 1 and the phantom data set respectively, for the various reconstruction and drift correction methods. Although the sensorless reconstructions exhibit no qualitatively obvious length error, there is a significant tilt error which is particularly obvious in the longer phantom sequence. Also, the fine scale accuracy of the sensorless reconstruction is noticeably superior to that of the position sensor reconstruction, particularly in the beef data, which is more susceptible to probe pressure jitter. Both of the drift correction methods are able to significantly reduce the tilt error, without over-fitting to position sensor noise and thereby sacrificing the fine scale accuracy. It is evident from these reslices that tilt bias is a more significant problem than length bias. The limited orientation correction therefore provides a worthwhile improvement, despite the fact that it is unable to correct length bias.

**Figure 9:**
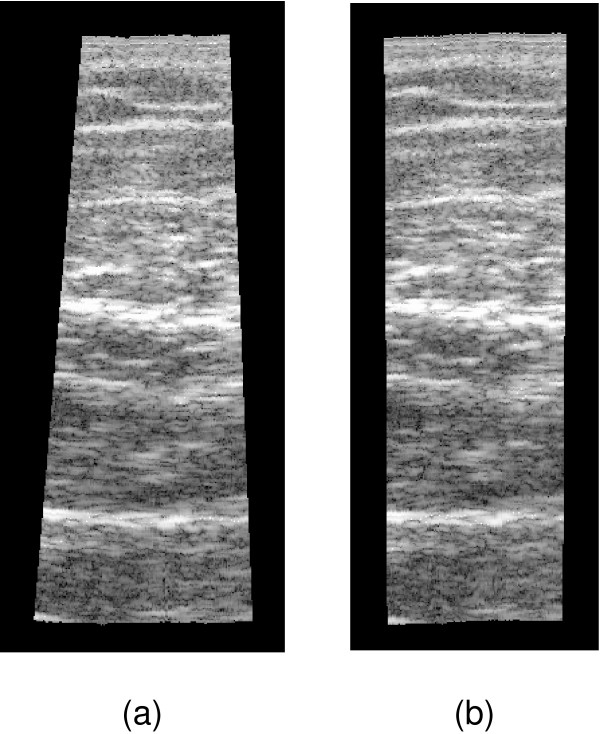
**Reslice images of human calf muscle**. The images are reslices along the length of the *in vivo *data set, showing the length and tilt. (a) Sensorless reconstruction. (b) Orientation-only correction using the MT9-B.

Figure 9 shows reslices through the *in vivo *calf muscle data set, corrected using the MT9-B. In this case, there are no six degree-of-freedom sensor readings for comparison. However, the data was recorded using an approximately linear scanning protocol, so we can be fairly confident that the drift corrected result using the MT9-B is a more accurate representation of the scanning subject than that obtained using purely sensorless techniques.

## Conclusion

Gold standard freehand 3D ultrasound acquisition, with accurate geometrical dimensions along with good fine scale detail, requires image-based reconstruction with six degree-of-freedom drift correction using a position sensor. Suitable position sensors, however, impose restrictions on the scanning protocol that make them somewhat obtrusive. A compromise is offered by the MT9-B orientation sensor. It is relatively unobtrusive and capable of correcting angular drift in sensorless reconstructions to within around 1°, although linear drift remains a problem. The MT9-B is easily calibrated using a variation of the established plane-based calibration method.

## Abbreviations

**2D **– two dimensional; **3D **– three dimensional; **4D **– four dimensional; **DOF **– degree of freedom; **MEMS **– Micro-Electro-Mechanical Systems

## Competing interests

The authors declare that they have no competing interests.

## Authors' contributions

RJH designed the MT9-B assessment protocol, developed the calibration disambiguation technique, designed the three degree-of-freedom sensorless drift correction algorithm and performed all the experiments. GMT designed the six degree-of-freedom sensorless drift correction algorithm. AHG and RWP suggested the MT9-B calibration protocol and designed aspects of the sensorless reconstruction algorithm. All authors contributed to the final text of the article.
